# Chemokines and microRNAs in atherosclerosis

**DOI:** 10.1007/s00018-015-1925-z

**Published:** 2015-05-23

**Authors:** Petra Hartmann, Andreas Schober, Christian Weber

**Affiliations:** 1 Institute for Cardiovascular Prevention, Ludwig-Maximilians-University (LMU) Munich, Munich, Germany; 2 DZHK (German Centre for Cardiovascular Research), Partner Site Munich Heart Alliance, Munich, Germany

**Keywords:** Chemokines, MicroRNAs, Atherosclerosis, Inflammation

## Abstract

The crucial role of chemokines in the initiation and progression of atherosclerosis has been widely recognized. Through essential functions in leukocyte recruitment, chemokines govern the infiltration with mononuclear cells and macrophage accumulation in atherosclerotic lesions. Beyond recruitment, chemokines also provide homeostatic functions supporting cell survival and mediating the mobilization and homing of progenitor cells. As a new regulatory layer, several microRNAs (miRNAs) have been found to modulate the function of endothelial cells (ECs), smooth muscle cells and macrophages by controlling the expression levels of chemokines and thereby affecting different stages in the progression of atherosclerosis. For instance, the expression of CXCL1 can be down-regulated by miR-181b, which inhibits nuclear factor-κB activation in atherosclerotic endothelium, thus attenuating the adhesive properties of ECs and exerting early atheroprotective effects. Conversely, CXCL12 expression can be induced by miR-126 in ECs through an auto-amplifying feedback loop to facilitate endothelial regeneration, thus limiting atherosclerosis and mediating plaque stabilization. In contrast, miR-155 plays a pro-atherogenic role by promoting the expression of CCL2 in M1-type macrophages, thereby enhancing vascular inflammation. Herein, we will review novel aspects of chemokines and their regulation by miRNAs during atherogenesis. Understanding the complex cross-talk of miRNAs controlling chemokine expression may open novel therapeutic options to treat atherosclerosis.

## Introduction

Chemokines are small chemotactic cytokines that play a crucial role in the trafficking of leukocytes by activating seven-transmembrane-domain G protein-coupled receptors (GPCRs) [1]. Studies using mouse models with genetic alteration in the chemokine-receptor system have highlighted a pivotal role of specific chemokine axes during different stages of atherosclerosis. Besides the structural classification based on their amino acid sequence, chemokines are grouped according to their function in homeostatic and inflammatory chemokines [2]. MicroRNAs (miRNAs) comprise a family of non-coding RNAs with a length of 22 nucleotides that act as post-transcriptional and cell type-specific repressors of gene expression [3, 4]. By regulating the expression of chemokines, miRNAs control various cellular processes implicated in atherosclerosis. The miRNA-mediated regulation of chemokines varies considerably between individual chemokines, which might allow an alternative classification system of chemokines. In the first part of the review, we will focus on chemokines and their role in atherosclerosis. In the following part, we will discuss miRNAs that control the expression of chemokines during different stages of atherosclerosis.

## Chemokines and chemokine receptors

Chemokines share a very similar tertiary structure, known as the typical chemokine fold, which is characterized by four cysteine residues that form two disulfide bridges [2]. At present, approximately 50 chemokines and more than 20 receptors have been identified in humans. Chemokines are classified into four families (CC, CXC, CX_3_C, and C motif chemokine) according to the configuration of the first two N-terminal cysteine residues [2]. Some chemokines can share several chemokine receptors within their families and vice versa various chemokine receptors can be activated by multiple chemokine ligands, as has been extensively reviewed and summarized in recent updates on the chemokine and chemokine-receptor superfamilies [5, 6]. In contrast, some receptor–ligand interactions are specific and monogamous such as CX_3_C motif chemokine 1 (CX_3_CL1) and its receptor CX_3_C chemokine receptor (CX_3_CR1) or CXCL16 and CXCR6. Almost all chemokines are secreted as soluble molecules except CX_3_CL1 and CXCL16, which are synthesized as transmembrane proteins and only released in a soluble form upon cleavage by metalloproteases [2].

Most chemokines can aggregate into oligomers, mainly forming homodimers, which can be divided into two classes. Whereas CC chemokines associate to elongated dimers through their N-termini, CXC chemokines dimerize to a globular quaternary structure [2]. In addition, chemokines form also heterodimers, as it is shown for CCL5 and CXCL4. Thereby, CXCL4 promotes the CCL5-mediated monocyte arrest on the endothelium [7, 8]. However, the activation of receptor occurs in the monomeric form at least in the context of cell migration. Studies with chemokine mutants that are unable to dimerize show that monomeric CCL2, CCL4, CCL5, CXCL1, and CXCL8 bind sufficiently to their receptors and induce cell migration in vitro [9–11]. In contrast, non-dissociating dimers of CXCL1 and CXCL8 can efficiently bind and activate CXCR2 but not CXCR1 [12, 13]. Although oligomerization is not required for receptor activation, it is essential for the function of some chemokines [9, 10]. Oligomerization may be important for the bridging presentation of chemokines bound to glycosaminoglycans (GAGs) to their receptor, since monomeric forms of CCL5 do not bind to heparin nor activate cell arrest [14–16]. In turn, GAGs can facilitate the oligomerization of chemokines, indicating that oligomerization and GAGs are closely linked [17]. Through GAG binding, chemokines may also form haptotactic gradients for recruitment of leukocytes. Blocking oligomerization and GAG binding of CCL5 inhibit leukocyte recruitment into atherosclerotic lesions [18], while CCL5 aggregation and binding to GAGs has been implicated in CCL5-induced apoptosis [19].

Homeostatic chemokines are constitutively expressed and navigate basal leukocyte trafficking during developmental processes, hematopoiesis or immune surveillance [6]. For instance, the CXCL12–CXCR4 axis exerts pivotal homeostatic function by mediating the homing and retention of hematopoietic stem and progenitor cells in the bone marrow [20]. In contrast, inflammatory chemokines are upregulated during inflammation, thus playing a crucial role in the innate and adaptive immune response by triggering leukocyte recruitment to sites of inflammation [21]. The expression of inflammatory chemokines is tightly regulated by pro- and anti-inflammatory transcription factors such as nuclear factor-κB (NF-κB) or the anti-inflammatory factors Krüppel-like factor (KLF) 4 and 2 [22–24]. Some chemokines can be assigned to both groups and are referred to as dual-function chemokines [21]. In addition to leukocyte recruitment, members of the CXC chemokines family including CXCL12 are important during angiogenesis [25]. CXC chemokines are divided based on the presence and absence of the glutamic acid-leucine-arginine (ELR) motif in their N-terminus. CXC chemokines harboring an ELR motif such as CXCL1 and CXCL8 promote angiogenesis, whereas non-ELR-CXC chemokines such as CXCL4 can inhibit angiogenesis by interfering with other chemokines or growth factor binding [25].

The expression of chemokine receptors differs considerably between subtypes of leukocytes [5, 6]. Whereas most leukocyte subsets express CXCR4, they do not express CXCR7, both of which are receptors for CXCL12 [26, 27]. The short-term regulation of chemokine receptor expression on the cell surface occurs via chemokine-induced receptor down-modulation, which is part of the desensitization process that allows cells to adapt their response to chemokine stimulation. Desensitization can occur via receptor inactivation or internalization followed by recycling or lysosomal degradation [28]. The binding of chemokines to GPRCs (in a two site model involving the N-loop and N-terminus of chemokines) activates hetero-trimeric G proteins, primarily the inhibitory Gα_i_ protein. This affects down-stream second messengers like calcium and cyclic adenosine monophosphate, which induce the activation of signaling pathways including NF-κB or mitogen-activated (MAP) kinase pathway [29]. Thereby, chemokines can induce cellular responses such as the activation of integrins, which mediate the adhesion of leukocytes.

In contrast to classical GPCRs, atypical chemokine receptors (ACKR) such as Duffy antigen receptor for chemokines (DARCs) do not activate the conventional G protein-mediated signaling cascades due to the lack of the intracellular G protein-binding motif but may instead signal through β-arrestin [30]. The ACKR can function as a scavenger by sequestering chemokines from the circulation and shaping their local availability in tissues. The ACKR have recently been classified in a new nomenclature [5, 30].

### Functional mechanisms of chemokines during atherosclerosis

Chemokines play an essential role during different stages of atherosclerosis, as studied by mouse models with genetic alteration in the chemokine-receptor system. The combined blocking of CCL2 and the receptors CX_3_CR1 and CCR5 markedly attenuated bone-marrow monocytosis and reduced circulating monocyte numbers, which correlated with a substantially reduced accumulation of macrophages within atherosclerotic lesions [31]; thus, CCL2, CX3CR1, and CCR5 appear to play an additive and independent role during atherosclerosis, indicating that chemokines might regulate various processes of the accumulation of monocyte-derived macrophages within atherosclerotic lesions [31].

During the initial stages of atherosclerotic plaques development, EC dysfunction triggers an increased expression and immobilization of CXCL1 on the cell surface, thereby inducing monocyte adhesion by activating integrins on leukocytes [32–34]. Monocytes can be divided into at least two different functional subsets referred to as classical or inflammatory Ly-6C^high^ and non-classical or resident Ly-6C^low^ monocytes, which differ in their expression of chemokine receptors, especially for CCR2 and CX_3_CR1 [35]. Whereas classical monocytes express high levels of CCR2 and low levels of CX_3_CR1, non-classical monocytes are low in CCR2 and high in CX_3_CR1 expression [35]. During atherogenesis, primarily classical monocytes adhere to the endothelium to contribute to lesion development, likely due to their increased mobilization into the circulation during hyperlipidemia, which is mediated by the CCL2/CCR2 axis (already under steady state) and by the CXCL1/CXCR2 axis [31, 36, 37]. The subsequent recruitment of circulating classical monocytes into the lesions is driven by the CCL5 receptors CCR1 and CCR5 [35]. In contrast to CCL2 which might not particularly contribute to the adhesion of monocytes, CCL5 deposited on atherosclerotic endothelium from platelet granules triggers pro-atherogenic monocyte arrest, an effect which is enhanced by its heteromeric interaction with CXCL4 [7, 8, 32, 38]. Adherent monocytes transmigrate into the subendothelial space triggered by CCL2, where they differentiate into macrophages [32]. Besides a de novo influx of monocytes, the local proliferation of macrophages plays a dominant role in lesional macrophage accumulation [39]. Beyond a role in platelet-monocyte complex formation and recruitment [40], the CX_3_CL1–CX_3_CR1 axis protects against apoptosis and enforces the survival of monocytes and macrophages in the lesions, thus exerting an important homeostatic function in atherosclerosis [41]. In the intima, macrophages engulf modified lipoproteins and accumulate cholesterol intracellular, thereby developing into foam cells [42]. Excessive intracellular lipid storage induces the inflammatory response of macrophages characterized by the secretion of cytokines and chemokines such as CCL2, thus promoting the ongoing chronic inflammatory process [42, 43]. During the progression of atherosclerosis, smooth muscle cells (SMCs) migrate from the media to the intima of arteries, where they proliferate and secrete extracellular matrix components such as collagen. Thereby, SMCs build up a fibrous cap, which increases the stability of atherosclerotic lesions against rupture [44]. Notably, silencing the CXCL12-CXCR4 axis promotes the stability of atherosclerotic lesion by increasing the lesional SMC accumulation, due to an enhanced smooth muscle progenitor cell (SPC) recruitment into the lesions [45].

## Biogenesis and function of miRNAs

MicroRNAs are presumed to regulate most of the human protein-coding genes, given that over 60 % of human protein-coding, genes are conserved targets of miRNAs [46]. Most of the miRNAs are processed by the RNA polymerase II from either independent genes or located in introns of host genes as capped and polyadenylated primary transcripts (pri-miRs) containing a stem-loop structure [47]. The canonical miRNA maturation pathway comprises two cleavage steps, which involve type III RNase enzymes. In the nucleus, the microprocessor complex comprising the RNase III enzyme Drosha and its co-factor DGCR8 cleaves the pri-miRNA into a precursor miRNA hairpin (pre-miRNA), which is transported to the cytoplasm via exportin-5 [47]. The second RNase III enzyme Dicer cleaves the hairpin structure of pre-miRNAs near their terminal loop, which gives rise to a 22nt miR:miR* hetero-duplex. Dicer often interacts with double-stranded RNA-binding domain (dsRBD) proteins such as TAR-RBD proteins in humans, although these associations are not necessarily required for efficient Dicer-mediated miRNA processing [47]. Subsequently, the RNA duplex associates with argonaute proteins in an ATP-dependent process, thereby forming the pre-RNA-induced silencing complex (pre-RISC) [47]. During the maturation of the RISC, the miRNA duplex unwinds and one strand remains incorporated in the mature RISC (guide strands and miR), whereas the other strand (passenger strand or miR*) is released and usually degraded [47]. Strand selection does not always follow specific rules; however, the strand with the less thermodynamic stability at its 5′ end commonly remains in the RISC [47]. Not only the often abundant guide strand, but also the passenger strand can be selected to regulate the expression levels of its target genes [48].

Besides the canonical miRNA biogenesis pathway, miRNAs can be processed independently of Dicer or Drosha, like miR-451 and miR-320 [47]. The guide strand in the RISC regulates gene expression by base pairing of the seed sequence (nucleotide 2–8 in the 5′ end of the miR) and the miR response elements (MRE), which is predominantly located in the 3′ untranslated region (3′ UTR) of the target mRNA. Thereby, miRNAs inhibit gene expression by transcript destabilization or translational repression [47]. Beyond the canonical base-pairing pattern, miRNAs can also operate by binding to the 5′ UTR or protein-coding region of the target mRNA [49]. In addition to protein-coding transcripts, miRNA are occasionally found to target long-non-coding RNAs and ribosomal RNAs, transfer RNAs or small nuclear RNAs [49]. In human cells, 18,500 miRNA–mRNA interactions have been identified conferring a complex regulatory network [49]. Therefore, it is conceivable that the temporal and tissue-specific expression of chemokines is also controlled by miRNA interactions.

**Fig. 1:**
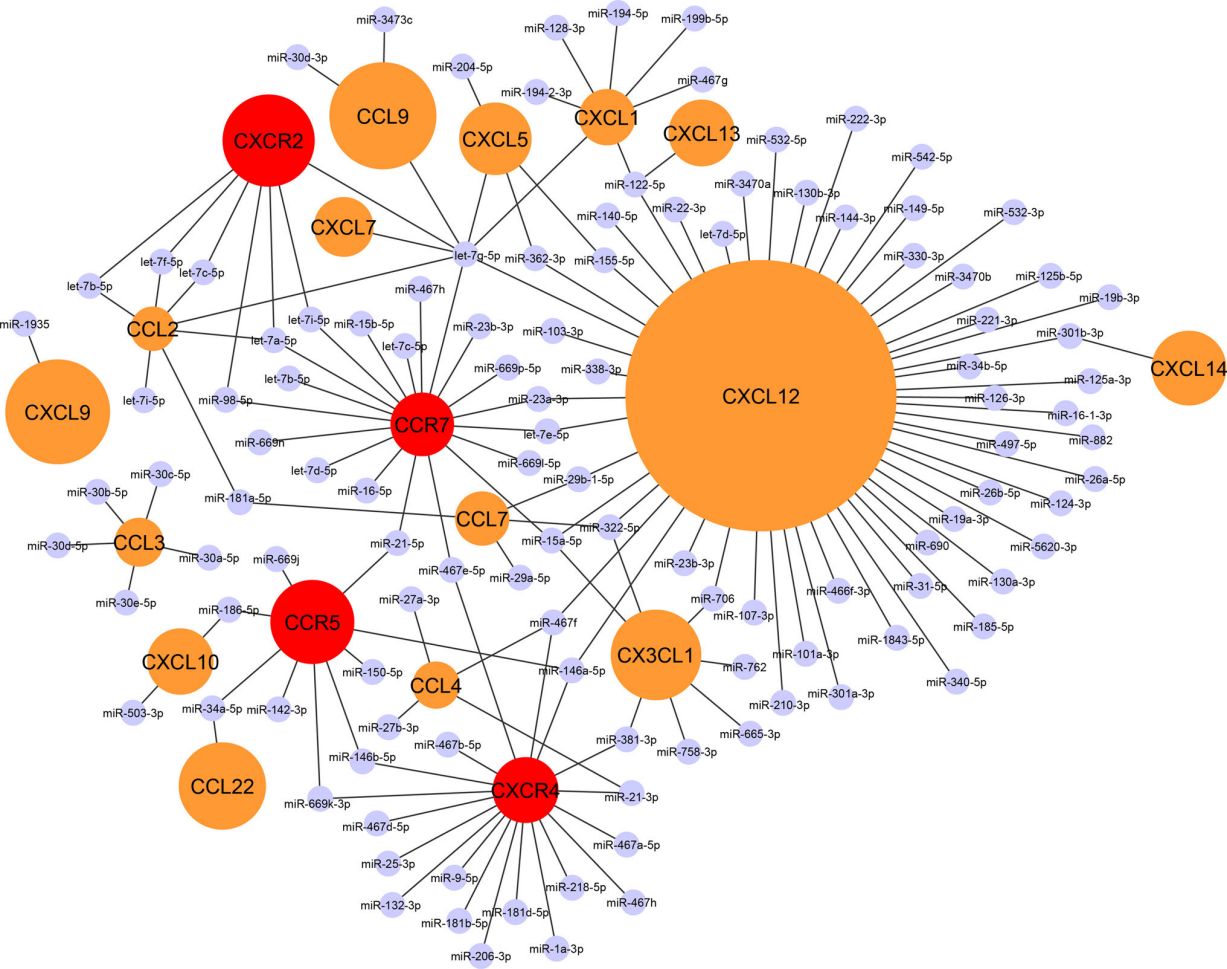
The chemokine–miRNA interactome: a classification of chemokines according to their miRNA-regulation pattern. *Black lines* are indicating experimentally validated interactions of miRNAs within the 3′ UTR region of murine chemokine or chemokine receptor transcripts using the web tool DIANA-TarBase v7.0 [109]. Chemokines/chemokine receptors without validated miRNA interactions have not been included in the interactome. The size of the *circles* in *orange* (chemokines) or *red* (chemokine receptors) corresponds to the number of predicted miRNA-binding sites in the 3′ UTR of the respective transcripts. A substantial number of putative miRNA-binding sites is predicted for CXCL12, which is in accordance with a high number of experimentally validated miRNA–CXCL12 interactions. In fact, a striking majority (53 %) of all validated miRNA interactions among all chemokines were observed for CXCL12 alone. In contrast, a limited number of miRNA interactions is predicted for other chemokines such as CCL2 or CXCL1 and for chemokine receptors, in accordance with a low number of functionally validated miRNA-binding sites for those transcripts

Notably, the number of predicted as well as experimentally validated miRNA interactions varies considerably between individual chemokines or chemokine receptors (Fig. 1), which might be due to the differences in their 3′ UTR length. For instance, CCL2 comprises a 3′ UTR length of only 373 base pairs, which might explain the low number of predicted miRNA-binding sites for CCL2. Accordingly, this limited number of MREs correlates with a low number of experimentally validated miRNA interactions (Fig. 1). In contrast, the 3′ UTR length of the homeostatic chemokine CXCL12 comprises 2630 base pairs, which correlates to a high number of predicted and validated miRNA interactions (Fig. 1). In fact, a vast majority of all miRNA–chemokine interactions either predicted (20 %) and validated (53 %) for chemokines are observed for CXCL12. Whereas the number of validated miRNA-bindings sites reveals marked differences between individual chemokines, chemokine receptors appear to comprise a more evenly distributed miRNA-controlled regulation pattern, given similar numbers of predicted and validated MREs (Fig. 1). Together with CCR7, the CXCL12 receptor CXCR4 still featured the highest number of validated miRNA interactions (19). Overall, the distinctive miRNA-mediated regulation patterns of chemokines might even allow for creating an alternative classification of chemokines, beyond previous structural and functional categories. In the subsequent chapters, we will focus on miRNAs that have been experimentally identified to regulate chemokine expression during different stages of atherosclerosis.

## CXCL1/CXCR2 axis and atherogenic monocyte arrest

The CXCR2 ligand CXCL1 is constitutively expressed in ECs within non-Weibel–Palade bodies and rapidly released upon stimulation with modified lipoproteins or lysophosphatidic acids (LPA), as part of an acute inflammatory cell response [34, 50]. By binding to heparin sulfate proteoglycans, CXCL1 is immobilized at the EC surface, thereby not only promoting the mobilization of monocytes from the bone marrow but also their adhesion to ECs by activating the β1-integrin VLA-4 [32, 33, 51]. In addition to the secretory release, endothelial activation transcriptionally increases the CXCL1 mRNA levels [34]. Inhibition of the LPA-mediated CXCL1 release impairs monocyte adhesion and atherosclerosis in apolipoprotein E-deficient mice [34]. Accordingly, the CXCL1–CXCR2 axis has been found to promote macrophage accumulation in established atherosclerotic lesions [52].

**Fig. 2:**
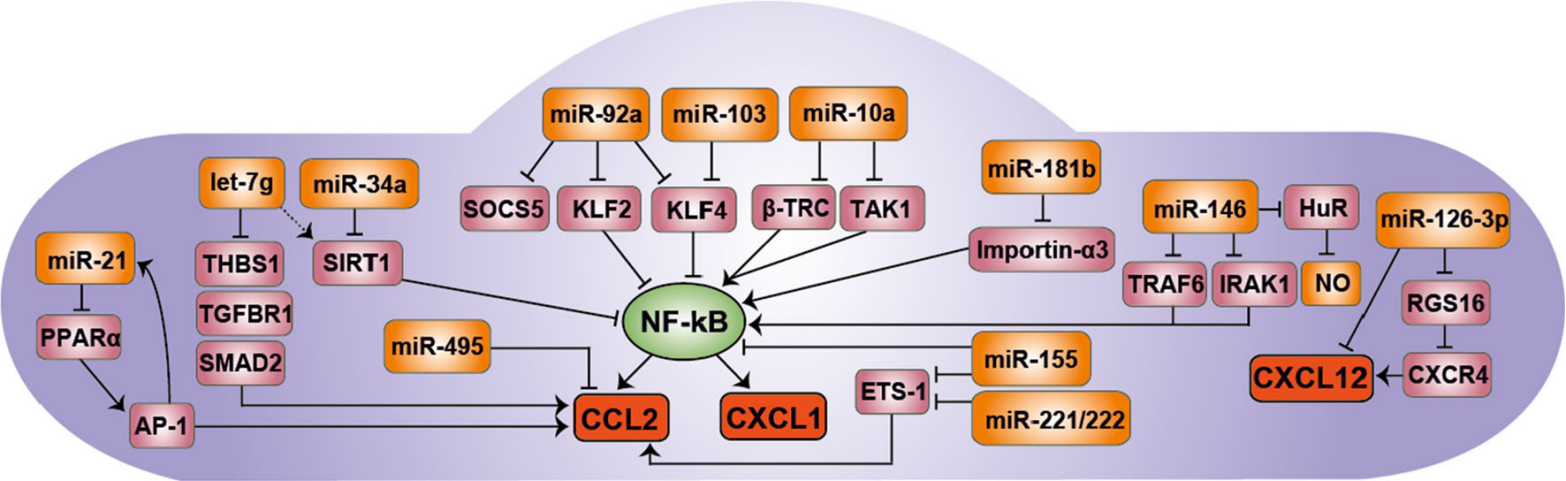
miRNAs control inflammatory response in ECs. miRNAs control the expression of inflammatory chemokines such as CCL2 and CXCL1 predominantly indirectly by regulating the expression of signaling molecules of the NF-κB signaling pathway. For instance, miR-181b inhibits the NF-κB-mediated CXCL1 expression by suppressing the expression of importin-α3, which is required for the nuclear translocation of NF-κB. miR-103 and miR-92a targets the NF-κB inhibitor KLF4, thereby increasing CCL2 and CXCL1 expression in ECs. In contrast, miR-21 reduces CCL2 expression by blocking the AP-1 signaling pathway. A direct regulation of CCL2 is reported for miR-495, which induces CCL2 mRNA degradation by binding to its response element in the 3′ UTR. Moreover, miR-126-3p controls the expression of CXCL12, directly and indirectly via its target RGS16. *NF-κB* nuclear factor-κB, *CCL2* chemokine (CC motif) ligand 2, *CXCL1* chemokine (CXC motif) ligand 1, *CXCL12* chemokine (CXC motif) ligand 12, *CXCR4* chemokine (CXC motif) receptor 4, *KLF2/4* Krüppel-like factor 2/4, *SOCS1* suppressor of cytokine signaling 1, *β‐TRC* β‐transducin repeat‐containing gene, *TAK1* transforming growth factor β-activated kinase 1, *TRAF6* TNF receptor-associated factor 6, *IRAK1* interleukin-1 receptor-associated kinase 1/2, *RGS16* regulator of G-protein signaling 16, *SIRT1* sirtuin-1, *THBS1* thrombospondin 1, *TGFBR1* transforming growth factor, beta receptor 1, *SMAD2* SMAD family member 2, *PPARα* peroxisome proliferator-activated receptor α, *AP-1* activator-protein 1. The *dashed arrow* indicates an indirect regulation

Disruption of miRNA biogenesis in ECs by endothelial Dicer deletion reduced the expression of CXCL1 in the arteries of apolipoprotein E-deficient mice, thereby attenuating monocyte adhesion to early atherosclerosis-prone endothelium [53]. TNF-α and hyperlipidemia induced the expression of miR-103, whereas the absence of endothelial Dicer consistently reduced miR-103 at different stages of atherosclerosis (Hartmann et al., unpublished data). In vitro, miR-103 promoted the expression and release of endothelial CXCL1 by translational repression of KLF4 through a conserved binding site in its 3′ UTR region (Table 1; Fig. 2). The expression of CX_3_CL1 and CCL2 was likewise regulated by the miR-103-mediated KLF4 suppression. KLF4 limits the activation of ECs by competing with NF-κB for binding to the coactivator p300. The impaired biogenesis of miR-103 expression following endothelial Dicer knock-out may contribute to reduced lesion formation and macrophage accumulation by balancing the functional antagonism between KLF4 and NF-κB. In addition, endothelial miR-92a can target both KLF4 and KLF2, thereby activating NF-κB signaling and the adhesion of monocytes to ECs [54, 55]. Although the effects of miR-92a on CXCL1 have not been studied, it seems reasonable to speculate that the miR-92a-mediated reduced monocyte adhesion is regulated via CXCL1 (Fig. 2). Notably, deficiency of KLF2 in macrophages accelerates atherosclerosis in hypercholesterolemic mice by increasing monocyte adhesion and macrophage infiltration into atherosclerotic lesions [56]. The expression of CXCL1 in peritoneal macrophages and plasma levels of CXCL1 is increased in mice harboring KLF2-deficient myeloid cells [57]. Functional studies showed that miR-150 mediates the KLF2-mediated CXCL1 suppression. However, the predicted miR-150 binding site in the 3′ UTR of CXCL1 mRNA has not been experimentally validated [57].

**Table 1:** Overview of miRNA-regulated chemokines in atherosclerosis

miRNA	Target	Effector cell	Affected chemokine	Role in atherosclerosis	References
miR-181b	Importin-α3	ECs	CXCL1	Protective	[58]
miR-103	KLF4	ECs	CXCL1 CCL2	Damaging	Unpublished
miR-126-3p	RGS16	ECs	CXCL12	Protective	[64]
miR-10a	TAK1, β‐TRC	ECs	CCL2	Protective	[87]
miR-92a	KLF2, KLF4, SOCS5	ECs	CCL2 CXCL1?	Damaging	[88]
miR-21	PPARα	ECs	CCL2	Damaging	[79]
let-7g	THBS1, TGFBR1, SMAD2	ECs	CCL2	Protective	[97]
miR-155	BCL6	Macrophages	CCL2	Damaging	[43]
miR-342-5p	AKT1	Macrophages	CCL2 (via miR-155)	Damaging	[102]
miR-467b	LPL	Macrophages	CCL2	Protective	[105]
miR-24	CHI3L1	Macrophages	CCL2	Protective	[108]

*miR/miRNA* microRNA, *RGS16* regulator of G-protein signaling 16, *TAK1* transforming growth factor β-activated kinase 1, *β‐TRC* β‐transducin repeat‐containing gene, *KLF2/4* Krüppel-like factor 2/4, *SOCS5* suppressor of cytokine signaling 5, *TLR4* toll-like receptor 4, *TRAF6* TNF receptor-associated factor 6, *IRAK1/2* interleukin-1 receptor-associated kinase 1/2, *PPARα* peroxisome proliferator-activated receptor α, *THBS1* thrombospondin 1, *TGFBR1* transforming growth factor, beta receptor 1, *SMAD2* SMAD family member 2, *Bcl6* B-cell lymphoma 6 protein, *Akt1* v-akt murine thymoma viral oncogene homolog 1, *CHI3L1* chitinase 3-like 1, *LPL* lipoprotein lipase

In contrast to miR-103, the expression levels of miR-181b are down-regulated in the aortic intima of mice treated with TNF-α or fed a high-fat diet, thereby enhancing CXCL1 expression (Table 1; Fig. 2) [58, 59]. The anti-inflammatory function of miR-181b was attributed to the inhibition of NF-κB by targeting the transporter protein importin-α3, which results in reduced nuclear translocation of p65 [58]. In vitro, miR-181b attenuates the adhesion of THP-1 cells to TNF-α stimulated ECs, most likely by reducing CXCL1 [58]. Systemic delivery of miR-181b limits NF-κB activation and inflammatory gene expression in the aortic intima of the vessel wall of apolipoprotein E-deficient mice. In addition, miR-181b overexpression reduces atherosclerotic lesion formation and macrophage accumulation in these mice by targeting importin α3 in aortic ECs [59]. Correspondingly, miR-181b levels are reduced in the plasma from patients with coronary artery disease. However, miR-181b does not regulate NF-κB signaling in leukocytes based on the distinct expression pattern of importin family members. In contrast to ECs, importin α5 was predominantly expressed in leukocytes.

Moreover, the adhesive properties of ECs are regulated by miR-712 and its human homolog miR-205 most likely by targeting the tissue metallopeptidase inhibitor 3 (TIMP3) in ECs, thereby triggering the release of soluble TNF-α. miR-712 increases monocyte adhesion in vitro and silencing of miR-712 reduces atherosclerosis in mice [60]. Like for miR-92a, the effects of miR-712 on monocyte adhesion may be indicative of a CXCL1-mediated response; however, a causal relationship has not been investigated in this study [60].

## Importance of CXCL12/CXCR4 axis in vascular repair

In atherosclerotic lesions, CXCL12 and its receptor CXCR4 are highly expressed in SMCs, ECs, and macrophages, but not in normal vessels [61]. CXCL12 plays an important role in homing and mobilization of progenitor cells that express CXCR4 on their surface [62]. During atherosclerosis, CXCR4 plays a protective role by controlling neutrophil mobilization and homeostasis [63]. Moreover, silencing of CXCL12 in the vessel wall of CXCL12-treated mice increases lesion formation with a reduced SMC content, corroborating an anti-atherogenic effect of the CXCL12–CXCR4 axis [45]. Notably, CXCL12 treatment in apolipoprotein E-deficient mice elevates CXCL12 plasma levels and promotes the transcription of CXCL12 in ECs [45]. Accordingly, endothelial CXCL12 expression is auto-amplified via a CXCR4-mediated feedback loop unleashed by miR-126-3p, which is delivered by endothelial apoptotic bodies (Table 1; Fig. 2) [64]. Thereby, miR-126-3p suppresses its target RGS16, a negative regulator of G-protein-dependent CXCR4 signaling. The upregulation of endothelial CXCL12 mediated by apoptotic body-derived miR-126-3p in early atherosclerosis reduces lesion formation by diminishing the lesional macrophage content and increasing SMC content in a CXCR4-dependent process [64]. Besides the CXCL12/CXCR4 axis, endothelial microparticle-derived miR-126-3p can also enhance endothelial repair by increasing the VEGF signaling through down-regulation of its target Sprouty-related EVH1 domain-containing protein, which acts as a VEGF signaling inhibitor [65]. However, the anti-atherogenic mechanisms of endothelial CXCL12 and CXC4 remain to be further clarified. Notably, the deficiency of endothelial CXCR4 in apolipoprotein E-deficient mice impairs re-endothelialization and endothelial progenitor cell recruitment after vascular injury, indicating that the CXCL12–CXCR4 axis in ECs exerts an atheroprotective role by inducing EC proliferation [66].

Interestingly, miR-126-3p can also directly target CXCL12 through a canonical binding site in the 3′ UTR region of CXCL12 mRNA, diminishing the recruitment of angiogenic progenitor cells to ischemic tissue [67]. The precursor of miR-126 forms two mature miRNAs, the guide strand miR-126-3p and the passenger strand miR-126-5p, which are derived from the 3′ arm and 5′ arm of the precursor miRNA, respectively. Both are functional active and target distinct or identical mRNAs. Likewise and synergistically with miR-126-3p, the passenger strand miR-126-5p can directly target CXCL12, thereby impairing inflammatory monocyte recruitment into the tumor stroma by reducing CCL2 expression [68]. Thus, the mechanism of mRNA target selection seems to be strongly dependent on the cell type and context. In fact, the interaction of miR-126-3p or miR-126-5p with CXCL12 does not play a significant role in the context of atherosclerosis. In this context, miR-126-5p rather targets delta-like 1 homolog, thereby promoting EC proliferation and limiting atherosclerosis [48].

Similar to microparticle-mediated delivery of miR-126-3p [64], systemic application of CXCL12 improves plaque stability as characterized by increasing SMC accumulation, collagen content, and fibrous cap thickness; however, it does not affect lesion size in apolipoprotein E-deficient mice with advanced atherosclerotic lesions [45]. The increased lesion stability was related to inducing the mobilization and recruitment of bone-marrow-derived SPCs into atherosclerotic lesions, where they can differentiate into SMC-like cells [45]. Similarly, extensive mobilization of SPCs occurs during vascular repair of injured arteries triggered by increased apoptosis of medial SMCs [69]. In response to apoptosis, LPA-induced CXCL12 is immobilized to vessel wall adherent-platelets, which promotes neointima formation through SPC recruitment, thereby mediating the repair of the vasculature [70–72]. Disruption of the CXCL12/CXCR4 axis after vascular injury by CXCR4 antagonists or in CXCR4-deficient bone-marrow cells limits neointima formation and SMC accumulation by reducing SPC recruitment [71, 73, 74]. In contrast to its protective effect in ECs during atherosclerosis, the upregulation of CXCL12 in neointimal cells during vascular repair accelerates neointima formation, indicating context- and cell type-specific roles for the CXCL12-CXCR4 axis in arterial diseases.

In addition to CXCR4, CXCL12 can also bind to its alternative receptor CXCR7. Whereas the mRNA transcripts of CXCR7 are highly expressed in the carotid artery wall, expression of CXCR7 protein is not detectable [75]. The discrepancy of CXCR7 mRNA and protein expression pattern suggests a translational repression by miRNAs, e.g., via miR-430, which directly targets CXCR7 [76]. Activation of CXCR7 in apolipoprotein E-deficient mice by a synthetic ligand lowers serum cholesterol levels under hyperlipidemic stress by promoting the receptor-dependent uptake of VLDL into adipose tissue. The lipid-lowering effect of CXCR7 limits atherosclerotic lesion formation by decreasing macrophage accumulation [75].

## Role of the CCL2/CCR2 axis in ECs and macrophages

The implication of chemokines in the pathogenesis of atherogenesis has been shown first for CCL2 and its receptor CCR2. Macrophages and ECs are a major source for CCL2 in human atherosclerotic lesions, whereas normal arteries do not express CCL2 [77, 78]. Stimuli such as hyperlipidemia, disturbed flow or angiotensin II induce the expression of CCL2 during atherosclerosis [79–81]. CCR2 is highly expressed on classical monocytes compared to non-classical monocytes, thereby defining the pro-inflammatory monocyte subtype [35].

Deficiency of CCL2 or CCR2 in apolipoprotein E-deficient mice or low-density lipoprotein (LDL) receptor-deficient mice attenuates the formation of atherosclerotic and neointimal lesions, reducing lesional macrophage content [82–84]. Accordingly, local overexpression of CCL2 at the vessel wall in combination with hyperlipidemia accelerates atherosclerosis by promoting the infiltration of macrophages into atherosclerotic lesions [85]. In contrast to CXCL1, which mediates monocyte adhesion by immobilization at the cell surface, CCL2 primarily contributes to the transmigration of adherent monocytes to the subendothelial space [32, 33]. Moreover, CCR2 deficiency in mice revealed an essential role for the CCL2/CCR2 axis in the mobilization of classical monocytes from the bone marrow to sites of inflammation, which reduces the number of circulating monocytes [37, 86]. Although the number of circulating monocytes correlates with the development of atherosclerotic plaques and with the content of monocytes/macrophages in the lesions [36], the causal relationship between CCL2-mediated monocytosis and accelerated atherosclerosis warrants further clarification.

### Control of CCL2 expression by miRNAs in ECs

Disturbed shear stress promotes the expression of CCL2 in ECs by suppressing miR-10a [87]. Inhibition of endothelial miR-10a activates NF-κB signaling by restoration of β‐transducin repeat‐containing gene (β‐TRC) and transforming growth factor β-activated kinase 1 (TAK1), both of which promote NF-κB activity (Table 1; Fig. 2). TAK1 directly phosphorylates and activates the IkB kinase β, which phosphorylates the inhibitor protein IkBα to trigger ubiquitination and proteasomal degradation through a complex including β‐TRC. Thus, both miR-10a targets induce the nuclear translocation of p65. Moreover, disturbed flow in combination with oxidized LDL increases the release of endothelial CCL2 by suppressing miR-92a expression levels in a STAT3-dependent manner [54]. By directly targeting KLF2 and KLF4, miR-92a reduces NF-κB-mediated CCL2 release [55, 88, 89]. In addition, CCL2 expression is attenuated by miR-92a-mediated repression of the target suppressor of cytokine signaling 5 (SOCS5), limiting endothelial inflammation (Table 1; Fig. 2). Although the functional relevance is still unknown, SOCS5 reduces the activation of the JAK-STAT pathway in ECs [90]. Notably, inhibition of miR-92a by antagomiR treatment reduces atherosclerotic lesion formation at the aortic arch in LDL receptor-knock-out mice. This is associated with lower lesional macrophage accumulation but increased collagen content, indicating that miR-92a promotes a more stable plaque phenotype [54]. Moreover, miR-92a suppresses endothelial proliferation after vascular injury, thereby disturbing endothelial repair. In atherosclerosis, insufficient EC proliferation accelerates the disease progression [48]. Notably, CCL2 inhibits EC proliferation in vitro under the regulation of miR-495, indicating an alternative pro-atherogenic role for CCL2 in ECs beyond mediating leukocyte recruitment [91] and triggering EC migration for endothelial wound repair [92].

**Fig. 3:**
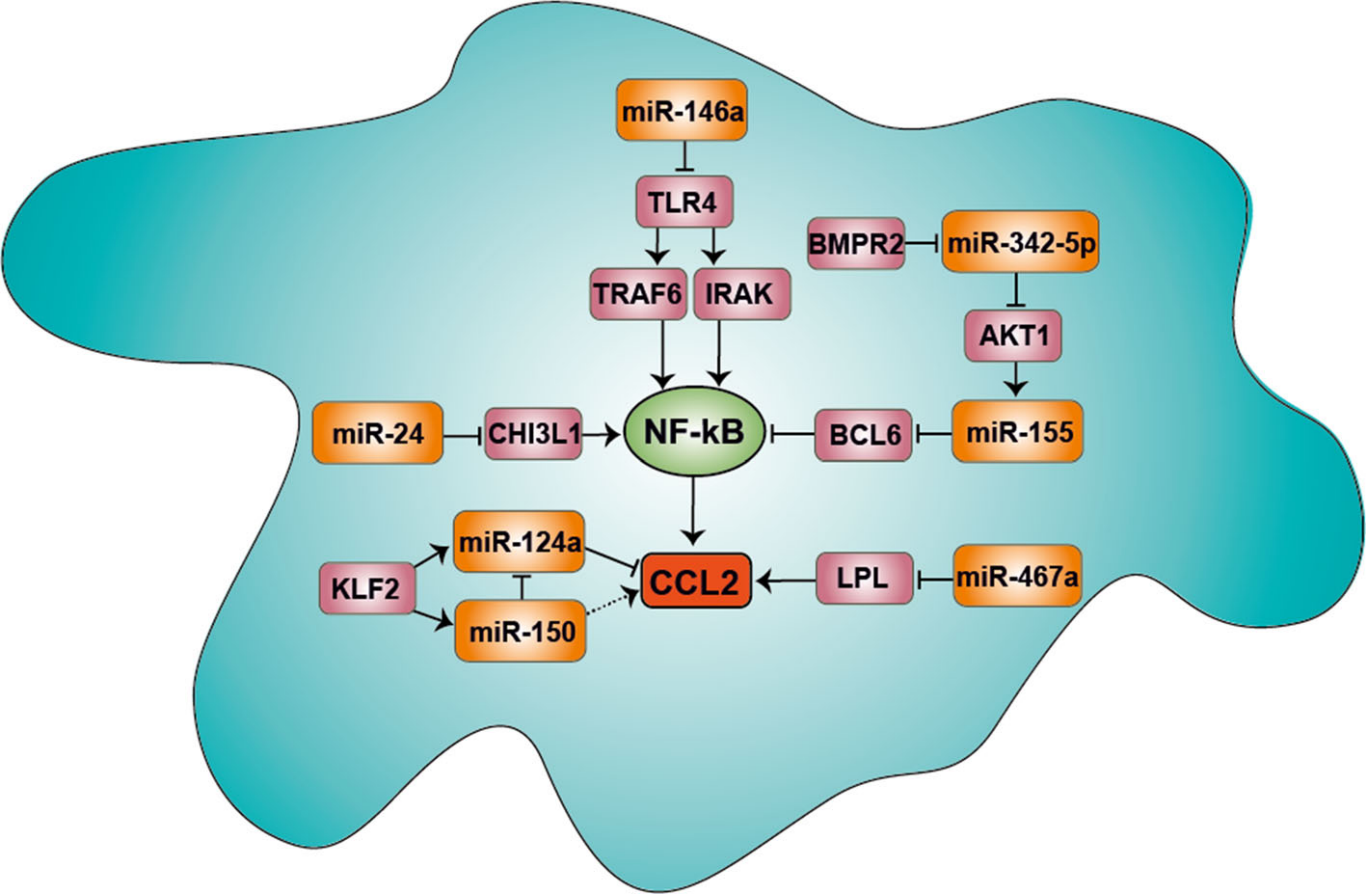
miRNA-mediated CCL2 expression in macrophages. The expression of CCL2 is induced by the miR-155-mediated suppression of the NF-κB inhibitor BCL6. miR-155 is regulated by miR-342-5p, which targets AKT-1 and BMPR2. miR-146 acts as a negative regulator to reduce the CCL2 expression by targeting TLR4. Moreover, CCL2 expression is triggered by the expression of CHI3L1 and LPL, which is negatively regulated by miR-24 and miR-467a, respectively. KLF2 expression in macrophages reduces the expression of CCL2 by reducing miR-150, which negatively regulates miR-124a. miR-124a directly suppresses the expression of CCL2 via its binding site in the 3′ UTR of the CCL2 mRNA transcript. *NF-κB* nuclear factor-κB, *CCL2* chemokine (CC motif) ligand 2, *BCL6* B-cell lymphoma 6, *AKT1* v-akt murine thymoma viral oncogene homolog 1, *BMPR2* bone morphogenetic protein receptor type II, *TLR4* toll-like receptor 4, *TRAF6* TNF receptor-associated factor 6, *IRAK1* interleukin-1 receptor-associated kinase 1/2, *CHI3L1* chitinase 3-like 1, *LPL* lipoprotein lipase, *KLF2* Krüppel-like factor 2. The *dashed arrow* indicates an indirect regulation

Notably, myeloid-specific deficiency of KLF2 in mice increases the expression of CCL2, likely by reducing miR-150 expression, which negatively regulates miR-124a targeting CCL2 (Fig. 3) [57, 93]. Accordingly, reduced miR-150 levels have been found to enhance the monocyte mobilization from the bone marrow [94]. Although the miR-150 effect in this study has been attributed to its target CXCR4, it is likely to additionally involve a CCL2-mediated response.

The induction of CCL2 by interleukin-1β (IL-1β) stimulation simultaneously elevates the expression of endothelial miR-146a/b, which targets TNF receptor-associated factor 6 (TRAF6) and IL-1 receptor-associated kinase (IRAK) 1/2 (Table 1; Fig. 2) [95]. TRAF6 and IRAK1/2 are adaptor proteins mediating IL-1β signaling pathways. Thereby, miR-146a/b functions as a negative regulator to restrain EC inflammation by repressing NF-κB activation as well as MAPK and activator-protein 1 (AP-1) pathways. In addition, miR-146 suppresses the RNA-binding protein HuR, which represses KLF2 as a transcriptional activator of eNOS. Treatment of miR-146 knock-out mice with IL-1β enhances CCL2 expression, likely due to a defect in the anti-inflammatory feedback loop, thereby inducing vascular inflammation [95].

In contrast, miR-21 sustains the oscillatory shear stress (OSS)-mediated CCL2 expression in ECs [79]. miR-21 targets the 3′ UTR of PPARα mRNA, leading to its translational suppression and activation of AP-1 signaling. Moreover, AP-1 promotes the OSS-induced expression of miR-21 and inflammatory activation, thus constituting a positive feedback loop, by which miR-21 is auto-amplified (Table 1; Fig. 2) [79]. Like miR-21, miR-34 acts as a regulator of OSS-mediated CCL2 expression by suppressing the NF-κB inhibitor sirtuin-1 in ECs [96]. Similarly, sirtuin-1 expression levels are indirectly increased by let-7g, thereby preventing endothelial senescence [97]. Moreover, let-7g diminishes CCL2 expression in ECs and monocyte adhesion by targeting three genes of the transforming growth factor (TGF)-β signaling pathway, thrombospondin 1, TGF-β receptor 1, and SMAD family member 2. Overexpression of let-7g prevents monocyte infiltration and CCL2 expression in carotid arteries of apolipoprotein E-deficient mice, whereas inhibition of let-7g using let-7g sponge plasmids increases macrophage accumulation (Fig. 2) [97]. Besides disturbed flow, angiotensin-II promotes CCL2 expression in ECs by activating the transcription factor Ets-1 [98]. Overexpression of miR-155 and miR-221/222 in ECs decreases angiotensin-II-induced CCL2 expression and T cell adhesion to ECs by targeting Ets-1 (Fig. 2) [99]. However, the causal relationship between CCL2 and monocyte adhesion has not been addressed in these studies.

### Control of CCL2 expression by miRNAs in macrophages

After transmigration into the intima, monocytes differentiate into macrophages, which further propagate inflammation by secreting CCL2. The expression of CCL2 in inflammatory macrophages is driven by miR-155, which is upregulated in macrophages of atherosclerotic lesions (Table 1; Fig. 3) [43]. miR-155 represses the expression of B-cell lymphoma 6 (Bcl6), a transcription factor attenuating NF-κB activation. Bcl6 maintains macrophage quiescence by transcriptional repression via histone deacetylation of enhancer regions from inflammatory genes [100]. Deficiency of Bcl6 in bone-marrow cells of LDL receptor-deficient mice substantially promotes atherosclerosis by increasing CCL2 expression [101]. Accordingly, apolipoprotein E-deficient mice harboring miR-155 knock-out bone marrow show reduced atherosclerotic lesion formation and macrophage content associated with Bcl6-mediated CCL2 suppression. miR-342-5p enhances the expression of miR-155 during the progression of atherosclerosis by suppressing the target v-akt murine thymoma viral oncogene homolog 1, which acts as an inhibitor of miR-155 [102]. Silencing of miR-342-5p in apolipoprotein E-deficient mice by locked nucleic acid (LNA) antisense oligonucleotides reduces macrophage accumulation, lesion formation as well as miR-155 and pro-inflammatory cytokine expression, indicating a miR-155-mediated inflammatory role of miR-342-5p (Table 1; Fig. 3) [102].

Whereas miR-155 primarily enhances CCL2 expression in macrophages, miR-146 acts as a negative regulator to restrain NF-κB-mediated CCL2 expression by targeting toll-like receptor 4 (TLR4) [103]. Notably, deficiency of TLR4 in apolipoprotein E-deficient mice is associated with lower CCL2 serum levels, reduced atherosclerosis and macrophage infiltration [104]. Likewise, miR-467b in macrophages protects apolipoprotein E-deficient mice from atherosclerosis, likely through decreasing both plasma and lesional CCL2 levels, and through suppression of lipoprotein lipase (Fig. 3) [105]. Moreover, the abundant expression and secretion of chitinase 3-like 1 (CHI3L1) at early stages of atherosclerosis triggers CCL2 expression in macrophages via activation of MAPK and NF-κB pathways [106]. CHI3L1 levels are elevated in patients with coronary artery disease and correlate with disease progression [107]. miR-24 negatively regulates the expression of CHI3L1, indicating an anti-inflammatory role of miR-24. Accordingly, inhibition of miR-24 using LNA oligonucleotides accelerates the atherosclerotic lesion formation and macrophage accumulation; however, it is not known, whether the atheroprotective effect of miR-24 is CCL2-dependent (Table 1; Fig. 3) [108].

## Conclusions

Over the recent years, increasing evidence has highlighted the crucial role for miRNAs as regulators of chemokines to modulate and control inflammatory processes during the initiation and progression of atherosclerosis. Overall, it is remarkable that miRNAs appear to regulate chemokines primarily through an indirect regulation of pro- or anti-inflammatory transcription factors, rather than directly via miRNA-binding sites in their mRNA transcripts. This might be due to the comparably short length of the 3′ UTR for chemokines, with CXCL12 representing one notable exception. It is conceivable that the ubiquitously important roles of CXCL12 in homeostasis versus disease require an elaborate, cell- and context-specific regulatory control of its expression that can be accomplished by miRNAs. Under conditions of inflammatory stimulation, the indirect regulation of chemokines further reflects the ability of miRNAs to fine-tune entire signaling cascades affecting the expression of more than one chemokine, which results in a more global and broad cellular response.

The treatment of inflammatory diseases by targeting the chemokine system is a challenging field due to the high risk of side effects such as impaired host defense against pathogens. Therefore, the specific and partial blockade of specific chemokine-receptor axes that are interacting together during atherosclerosis might be a promising therapeutic approach. Hence, context- and cell-specific effects of miRNA-target interactions may allow selective manipulations of the chemokine system during atherosclerosis without immunological side effects. For instance, current evidence suggests that the therapeutic application of miR-181b may limit the release of CXCL1 from the vascular endothelium and thereby reducing atherosclerosis, whereas the endothelial delivery of miR-126-3p boosts atheropotective CXCL12 expression. Moreover, blocking the interaction of miR-155 and its target Bcl6 maintains macrophage quiescence by suppressing CCL2 expression, which prevents the progression of atherosclerosis. It can be envisioned that further interactions of miRNAs with chemokines will emerge from future research to allow for combined and complementary targeting of chemokines through strategies employing miRNA mimetics or antagoMirs.

## References

[1] Zernecke A, Weber C (2014). Chemokines in atherosclerosis: proceedings resumed. Arterioscler Thromb Vasc Biol.

[2] Allen SJ, Crown SE, Handel TM (2007). Chemokine: receptor structure, interactions, and antagonism. Annu Rev Immunol.

[3] Nazari-Jahantigh M, Egea V, Schober A, Weber C (2014). MicroRNA-specific regulatory mechanisms in atherosclerosis. J Mol Cell Cardiol.

[4] Wei Y, Nazari-Jahantigh M, Neth P, Weber C, Schober A (2013). MicroRNA-126, -145, and -155: a therapeutic triad in atherosclerosis?. Arterioscler Thromb Vasc Biol.

[5] Bachelerie F, Ben-Baruch A, Burkhardt AM, Combadiere C, Farber JM, Graham GJ, Horuk R, Sparre-Ulrich AH, Locati M, Luster AD, Mantovani A, Matsushima K, Murphy PM, Nibbs R, Nomiyama H, Power CA, Proudfoot AE, Rosenkilde MM, Rot A, Sozzani S, Thelen M, Yoshie O, Zlotnik A (2014). International Union of Basic and Clinical Pharmacology [corrected]. LXXXIX. Update on the extended family of chemokine receptors and introducing a new nomenclature for atypical chemokine receptors. Pharmacol Rev.

[6] Zlotnik A, Yoshie O (2012). The chemokine superfamily revisited. Immunity.

[7] Koenen RR, von Hundelshausen P, Nesmelova IV, Zernecke A, Liehn EA, Sarabi A, Kramp BK, Piccinini AM, Paludan SR, Kowalska MA, Kungl AJ, Hackeng TM, Mayo KH, Weber C (2009). Disrupting functional interactions between platelet chemokines inhibits atherosclerosis in hyperlipidemic mice. Nat Med.

[8] von Hundelshausen P, Koenen RR, Sack M, Mause SF, Adriaens W, Proudfoot AE, Hackeng TM, Weber C (2005). Heterophilic interactions of platelet factor 4 and RANTES promote monocyte arrest on endothelium. Blood.

[9] Rajarathnam K, Sykes BD, Kay CM, Dewald B, Geiser T, Baggiolini M, Clark-Lewis I (1994). Neutrophil activation by monomeric interleukin-8. Science.

[10] Proudfoot AE, Handel TM, Johnson Z, Lau EK, LiWang P, Clark-Lewis I, Borlat F, Wells TN, Kosco-Vilbois MH (2003). Glycosaminoglycan binding and oligomerization are essential for the in vivo activity of certain chemokines. Proc Natl Acad Sci USA.

[11] Paavola CD, Hemmerich S, Grunberger D, Polsky I, Bloom A, Freedman R, Mulkins M, Bhakta S, McCarley D, Wiesent L, Wong B, Jarnagin K, Handel TM (1998). Monomeric monocyte chemoattractant protein-1 (MCP-1) binds and activates the MCP-1 receptor CCR2B. J Biol Chem.

[12] Ravindran A, Sawant KV, Sarmiento J, Navarro J, Rajarathnam K (2013). Chemokine CXCL1 dimer is a potent agonist for the CXCR2 receptor. J Biol Chem.

[13] Nasser MW, Raghuwanshi SK, Grant DJ, Jala VR, Rajarathnam K, Richardson RM (2009). Differential activation and regulation of CXCR1 and CXCR2 by CXCL8 monomer and dimer. J Immunol.

[14] Vives RR, Sadir R, Imberty A, Rencurosi A, Lortat-Jacob H (2002). A kinetics and modeling study of RANTES(9–68) binding to heparin reveals a mechanism of cooperative oligomerization. Biochemistry.

[15] Baltus T, Weber KS, Johnson Z, Proudfoot AE, Weber C (2003). Oligomerization of RANTES is required for CCR1-mediated arrest but not CCR5-mediated transmigration of leukocytes on inflamed endothelium. Blood.

[16] Das ST, Rajagopalan L, Guerrero-Plata A, Sai J, Richmond A, Garofalo RP, Rajarathnam K (2010). Monomeric and dimeric CXCL8 are both essential for in vivo neutrophil recruitment. PLoS ONE.

[17] Hoogewerf AJ, Kuschert GS, Proudfoot AE, Borlat F, Clark-Lewis I, Power CA, Wells TN (1997). Glycosaminoglycans mediate cell surface oligomerization of chemokines. Biochemistry.

[18] Braunersreuther V, Steffens S, Arnaud C, Pelli G, Burger F, Proudfoot A, Mach F (2008). A novel RANTES antagonist prevents progression of established atherosclerotic lesions in mice. Arterioscler Thromb Vasc Biol.

[19] Murooka TT, Wong MM, Rahbar R, Majchrzak-Kita B, Proudfoot AE, Fish EN (2006). CCL5-CCR5-mediated apoptosis in T cells: requirement for glycosaminoglycan binding and CCL5 aggregation. J Biol Chem.

[20] Doring Y, Pawig L, Weber C, Noels H (2014). The CXCL12/CXCR4 chemokine ligand/receptor axis in cardiovascular disease. Front Physiol.

[21] Moser B, Willimann K (2004). Chemokines: role in inflammation and immune surveillance. Ann Rheum Dis.

[22] Hamik A, Lin Z, Kumar A, Balcells M, Sinha S, Katz J, Feinberg MW, Gerzsten RE, Edelman ER, Jain MK (2007). Kruppel-like factor 4 regulates endothelial inflammation. J Biol Chem.

[23] Hajra L, Evans AI, Chen M, Hyduk SJ, Collins T, Cybulsky MI (2000). The NF-kappa B signal transduction pathway in aortic endothelial cells is primed for activation in regions predisposed to atherosclerotic lesion formation. Proc Natl Acad Sci USA.

[24] Gareus R, Kotsaki E, Xanthoulea S, van der Made I, Gijbels MJ, Kardakaris R, Polykratis A, Kollias G, de Winther MP, Pasparakis M (2008). Endothelial cell-specific NF-kappaB inhibition protects mice from atherosclerosis. Cell Metab.

[25] Mehrad B, Keane MP, Strieter RM (2007). Chemokines as mediators of angiogenesis. Thromb Haemost.

[26] Berahovich RD, Zabel BA, Penfold ME, Lewen S, Wang Y, Miao Z, Gan L, Pereda J, Dias J, Slukvin II, McGrath KE, Jaen JC, Schall TJ (2010). CXCR7 protein is not expressed on human or mouse leukocytes. J Immunol.

[27] Viola A, Luster AD (2008). Chemokines and their receptors: drug targets in immunity and inflammation. Annu Rev Pharmacol Toxicol.

[28] Fox JM, Letellier E, Oliphant CJ, Signoret N (2011). TLR2-dependent pathway of heterologous down-modulation for the CC chemokine receptors 1, 2, and 5 in human blood monocytes. Blood.

[29] Curnock AP, Logan MK, Ward SG (2002). Chemokine signalling: pivoting around multiple phosphoinositide 3-kinases. Immunology.

[30] Novitzky-Basso I, Rot A (2012). Duffy antigen receptor for chemokines and its involvement in patterning and control of inflammatory chemokines. Front Immunol.

[31] Combadiere C, Potteaux S, Rodero M, Simon T, Pezard A, Esposito B, Merval R, Proudfoot A, Tedgui A, Mallat Z (2008). Combined inhibition of CCL2, CX3CR1, and CCR5 abrogates Ly6C(hi) and Ly6C(lo) monocytosis and almost abolishes atherosclerosis in hypercholesterolemic mice. Circulation.

[32] Weber KS, von Hundelshausen P, Clark-Lewis I, Weber PC, Weber C (1999). Differential immobilization and hierarchical involvement of chemokines in monocyte arrest and transmigration on inflamed endothelium in shear flow. Eur J Immunol.

[33] Huo Y, Weber C, Forlow SB, Sperandio M, Thatte J, Mack M, Jung S, Littman DR, Ley K (2001). The chemokine KC, but not monocyte chemoattractant protein-1, triggers monocyte arrest on early atherosclerotic endothelium. J Clin Invest.

[34] Zhou Z, Subramanian P, Sevilmis G, Globke B, Soehnlein O, Karshovska E, Megens R, Heyll K, Chun J, Saulnier-Blache JS, Reinholz M, van Zandvoort M, Weber C, Schober A (2011). Lipoprotein-derived lysophosphatidic acid promotes atherosclerosis by releasing CXCL1 from the endothelium. Cell Metab.

[35] Gautier EL, Jakubzick C, Randolph GJ (2009). Regulation of the migration and survival of monocyte subsets by chemokine receptors and its relevance to atherosclerosis. Arterioscler Thromb Vasc Biol.

[36] Soehnlein O, Drechsler M, Doring Y, Lievens D, Hartwig H, Kemmerich K, Ortega-Gomez A, Mandl M, Vijayan S, Projahn D, Garlichs CD, Koenen RR, Hristov M, Lutgens E, Zernecke A, Weber C (2013). Distinct functions of chemokine receptor axes in the atherogenic mobilization and recruitment of classical monocytes. EMBO Mol Med.

[37] Tsou CL, Peters W, Si Y, Slaymaker S, Aslanian AM, Weisberg SP, Mack M, Charo IF (2007). Critical roles for CCR2 and MCP-3 in monocyte mobilization from bone marrow and recruitment to inflammatory sites. J Clin Invest.

[38] Mause SF, von Hundelshausen P, Zernecke A, Koenen RR, Weber C (2005). Platelet microparticles: a transcellular delivery system for RANTES promoting monocyte recruitment on endothelium. Arterioscler Thromb Vasc Biol.

[39] Robbins CS, Hilgendorf I, Weber GF, Theurl I, Iwamoto Y, Figueiredo JL, Gorbatov R, Sukhova GK, Gerhardt LM, Smyth D, Zavitz CC, Shikatani EA, Parsons M, van Rooijen N, Lin HY, Husain M, Libby P, Nahrendorf M, Weissleder R, Swirski FK (2013). Local proliferation dominates lesional macrophage accumulation in atherosclerosis. Nat Med.

[40] Postea O, Vasina EM, Cauwenberghs S, Projahn D, Liehn EA, Lievens D, Theelen W, Kramp BK, Butoi ED, Soehnlein O, Heemskerk JW, Ludwig A, Weber C, Koenen RR (2012). Contribution of platelet CX(3)CR1 to platelet-monocyte complex formation and vascular recruitment during hyperlipidemia. Arterioscler Thromb Vasc Biol.

[41] Landsman L, Bar-On L, Zernecke A, Kim KW, Krauthgamer R, Shagdarsuren E, Lira SA, Weissman IL, Weber C, Jung S (2009). CX3CR1 is required for monocyte homeostasis and atherogenesis by promoting cell survival. Blood.

[42] Weber C, Noels H (2011). Atherosclerosis: current pathogenesis and therapeutic options. Nat Med.

[43] Nazari-Jahantigh M, Wei Y, Noels H, Akhtar S, Zhou Z, Koenen RR, Heyll K, Gremse F, Kiessling F, Grommes J, Weber C, Schober A (2012). MicroRNA-155 promotes atherosclerosis by repressing Bcl6 in macrophages. J Clin Invest.

[44] Ross R (1999). Atherosclerosis—an inflammatory disease. N Engl J Med.

[45] Akhtar S, Gremse F, Kiessling F, Weber C, Schober A (2013). CXCL12 promotes the stabilization of atherosclerotic lesions mediated by smooth muscle progenitor cells in Apoe-deficient mice. Arterioscler Thromb Vasc Biol.

[46] Friedman RC, Farh KK, Burge CB, Bartel DP (2009). Most mammalian mRNAs are conserved targets of microRNAs. Genome Res.

[47] Ha M, Kim VN (2014). Regulation of microRNA biogenesis. Nat Rev Mol Cell Biol.

[48] Schober A, Nazari-Jahantigh M, Wei Y, Bidzhekov K, Gremse F, Grommes J, Megens RT, Heyll K, Noels H, Hristov M, Wang S, Kiessling F, Olson EN, Weber C (2014). MicroRNA-126-5p promotes endothelial proliferation and limits atherosclerosis by suppressing Dlk1. Nat Med.

[49] Helwak A, Kudla G, Dudnakova T, Tollervey D (2013). Mapping the human miRNA interactome by CLASH reveals frequent noncanonical binding. Cell.

[50] Oynebraten I, Bakke O, Brandtzaeg P, Johansen FE, Haraldsen G (2004). Rapid chemokine secretion from endothelial cells originates from 2 distinct compartments. Blood.

[51] Schwartz D, Andalibi A, Chaverri-Almada L, Berliner JA, Kirchgessner T, Fang ZT, Tekamp-Olson P, Lusis AJ, Gallegos C, Fogelman AM (1994). Role of the GRO family of chemokines in monocyte adhesion to MM-LDL-stimulated endothelium. J Clin Invest.

[52] Boisvert WA, Rose DM, Johnson KA, Fuentes ME, Lira SA, Curtiss LK, Terkeltaub RA (2006). Up-regulated expression of the CXCR2 ligand KC/GRO-alpha in atherosclerotic lesions plays a central role in macrophage accumulation and lesion progression. Am J Pathol.

[53] Suarez Y, Fernandez-Hernando C, Pober JS, Sessa WC (2007). Dicer dependent microRNAs regulate gene expression and functions in human endothelial cells. Circ Res.

[54] Loyer X, Potteaux S, Vion AC, Guerin CL, Boulkroun S, Rautou PE, Ramkhelawon B, Esposito B, Dalloz M, Paul JL, Julia PL, Maccario J, Boulanger CM, Mallat Z, Tedgui A (2013). Inhibition of microRNA-92a prevents endothelial dysfunction and atherosclerosis in mice. Circ Res.

[55] Fang Y, Davies PF (2012). Site-specific microRNA-92a regulation of Kruppel-like factors 4 and 2 in atherosusceptible endothelium. Arterioscler Thromb Vasc Biol.

[56] Lingrel JB, Pilcher-Roberts R, Basford JE, Manoharan P, Neumann J, Konaniah ES, Srinivasan R, Bogdanov VY, Hui DY (2012). Myeloid-specific Kruppel-like factor 2 inactivation increases macrophage and neutrophil adhesion and promotes atherosclerosis. Circ Res.

[57] Manoharan P, Basford JE, Pilcher-Roberts R, Neumann J, Hui DY, Lingrel JB (2014). Reduced levels of microRNAs miR-124a and miR-150 are associated with increased proinflammatory mediator expression in Kruppel-like factor 2 (KLF2)-deficient macrophages. J Biol Chem.

[58] Sun X, Icli B, Wara AK, Belkin N, He S, Kobzik L, Hunninghake GM, Vera MP, Blackwell TS, Baron RM, Feinberg MW (2012). MicroRNA-181b regulates NF-kappaB-mediated vascular inflammation. J Clin Invest.

[59] Sun X, He S, Wara AK, Icli B, Shvartz E, Tesmenitsky Y, Belkin N, Li D, Blackwell TS, Sukhova GK, Croce K, Feinberg MW (2014). Systemic delivery of microRNA-181b inhibits nuclear factor-kappaB activation, vascular inflammation, and atherosclerosis in apolipoprotein E-deficient mice. Circ Res.

[60] Son DJ, Kumar S, Takabe W, Kim CW, Ni CW, Alberts-Grill N, Jang IH, Kim S, Kim W, Won Kang S, Baker AH, Woong Seo J, Ferrara KW, Jo H (2013). The atypical mechanosensitive microRNA-712 derived from pre-ribosomal RNA induces endothelial inflammation and atherosclerosis. Nat Commun.

[61] Abi-Younes S, Sauty A, Mach F, Sukhova GK, Libby P, Luster AD (2000). The stromal cell-derived factor-1 chemokine is a potent platelet agonist highly expressed in atherosclerotic plaques. Circ Res.

[62] Schober A, Karshovska E, Zernecke A, Weber C (2006). SDF-1alpha-mediated tissue repair by stem cells: a promising tool in cardiovascular medicine?. Trends Cardiovasc Med.

[63] Zernecke A, Bot I, Djalali-Talab Y, Shagdarsuren E, Bidzhekov K, Meiler S, Krohn R, Schober A, Sperandio M, Soehnlein O, Bornemann J, Tacke F, Biessen EA, Weber C (2008). Protective role of CXC receptor 4/CXC ligand 12 unveils the importance of neutrophils in atherosclerosis. Circ Res.

[64] Zernecke A, Bidzhekov K, Noels H, Shagdarsuren E, Gan L, Denecke B, Hristov M, Koppel T, Jahantigh MN, Lutgens E, Wang S, Olson EN, Schober A, Weber C (2009). Delivery of microRNA-126 by apoptotic bodies induces CXCL12-dependent vascular protection. Sci Signal.

[65] Jansen F, Yang X, Hoelscher M, Cattelan A, Schmitz T, Proebsting S, Wenzel D, Vosen S, Franklin BS, Fleischmann BK, Nickenig G, Werner N (2013). Endothelial microparticle-mediated transfer of MicroRNA-126 promotes vascular endothelial cell repair via SPRED1 and is abrogated in glucose-damaged endothelial microparticles. Circulation.

[66] Noels H, Zhou B, Tilstam PV, Theelen W, Li X, Pawig L, Schmitz C, Akhtar S, Simsekyilmaz S, Shagdarsuren E, Schober A, Adams RH, Bernhagen J, Liehn EA, Doring Y, Weber C (2014). Deficiency of endothelial CXCR4 reduces reendothelialization and enhances neointimal hyperplasia after vascular injury in atherosclerosis-prone mice. Arterioscler Thromb Vasc Biol.

[67] van Solingen C, de Boer HC, Bijkerk R, Monge M, van Oeveren-Rietdijk AM, Seghers L, de Vries MR, van der Veer EP, Quax PH, Rabelink TJ, van Zonneveld AJ (2011). MicroRNA-126 modulates endothelial SDF-1 expression and mobilization of Sca-1(+)/Lin(−) progenitor cells in ischaemia. Cardiovasc Res.

[68] Zhang Y, Yang P, Sun T, Li D, Xu X, Rui Y, Li C, Chong M, Ibrahim T, Mercatali L, Amadori D, Lu X, Xie D, Li QJ, Wang XF (2013). miR-126 and miR-126* repress recruitment of mesenchymal stem cells and inflammatory monocytes to inhibit breast cancer metastasis. Nat Cell Biol.

[69] Schober A, Zernecke A (2007). Chemokines in vascular remodeling. Thromb Haemost.

[70] Schober A, Knarren S, Lietz M, Lin EA, Weber C (2003). Crucial role of stromal cell-derived factor-1alpha in neointima formation after vascular injury in apolipoprotein E-deficient mice. Circulation.

[71] Zernecke A, Schober A, Bot I, von Hundelshausen P, Liehn EA, Mopps B, Mericskay M, Gierschik P, Biessen EA, Weber C (2005). SDF-1alpha/CXCR4 axis is instrumental in neointimal hyperplasia and recruitment of smooth muscle progenitor cells. Circ Res.

[72] Subramanian P, Karshovska E, Reinhard P, Megens RT, Zhou Z, Akhtar S, Schumann U, Li X, van Zandvoort M, Ludin C, Weber C, Schober A (2010). Lysophosphatidic acid receptors LPA1 and LPA3 promote CXCL12-mediated smooth muscle progenitor cell recruitment in neointima formation. Circ Res.

[73] Karshovska E, Zagorac D, Zernecke A, Weber C, Schober A (2008). A small molecule CXCR4 antagonist inhibits neointima formation and smooth muscle progenitor cell mobilization after arterial injury. J Thromb Haemost.

[74] Hamesch K, Subramanian P, Li X, Dembowsky K, Chevalier E, Weber C, Schober A (2012). The CXCR4 antagonist POL5551 is equally effective as sirolimus in reducing neointima formation without impairing re-endothelialisation. Thromb Haemost.

[75] Li X, Zhu M, Penfold ME, Koenen RR, Thiemann A, Heyll K, Akhtar S, Koyadan S, Wu Z, Gremse F, Kiessling F, van Zandvoort M, Schall TJ, Weber C, Schober A (2014). Activation of CXCR7 limits atherosclerosis and improves hyperlipidemia by increasing cholesterol uptake in adipose tissue. Circulation.

[76] Liu L, Zhao X, Zhu X, Zhong Z, Xu R, Wang Z, Cao J, Hou Y (2013). Decreased expression of miR-430 promotes the development of bladder cancer via the upregulation of CXCR7. Mol Med Rep.

[77] Takeya M, Yoshimura T, Leonard EJ, Takahashi K (1993). Detection of monocyte chemoattractant protein-1 in human atherosclerotic lesions by an anti-monocyte chemoattractant protein-1 monoclonal antibody. Hum Pathol.

[78] Yla-Herttuala S, Lipton BA, Rosenfeld ME, Sarkioja T, Yoshimura T, Leonard EJ, Witztum JL, Steinberg D (1991). Expression of monocyte chemoattractant protein 1 in macrophage-rich areas of human and rabbit atherosclerotic lesions. Proc Natl Acad Sci USA.

[79] Zhou J, Wang KC, Wu W, Subramaniam S, Shyy JY, Chiu JJ, Li JY, Chien S (2011). MicroRNA-21 targets peroxisome proliferators-activated receptor-alpha in an autoregulatory loop to modulate flow-induced endothelial inflammation. Proc Natl Acad Sci USA.

[80] Liao F, Berliner JA, Mehrabian M, Navab M, Demer LL, Lusis AJ, Fogelman AM (1991). Minimally modified low density lipoprotein is biologically active in vivo in mice. J Clin Invest.

[81] Ni W, Kitamoto S, Ishibashi M, Usui M, Inoue S, Hiasa K, Zhao Q, Nishida K, Takeshita A, Egashira K (2004). Monocyte chemoattractant protein-1 is an essential inflammatory mediator in angiotensin II-induced progression of established atherosclerosis in hypercholesterolemic mice. Arterioscler Thromb Vasc Biol.

[82] Boring L, Gosling J, Cleary M, Charo IF (1998). Decreased lesion formation in CCR2^−/−^ mice reveals a role for chemokines in the initiation of atherosclerosis. Nature.

[83] Gu L, Okada Y, Clinton SK, Gerard C, Sukhova GK, Libby P, Rollins BJ (1998). Absence of monocyte chemoattractant protein-1 reduces atherosclerosis in low density lipoprotein receptor-deficient mice. Mol Cell.

[84] Schober A, Zernecke A, Liehn EA, von Hundelshausen P, Knarren S, Kuziel WA, Weber C (2004). Crucial role of the CCL2/CCR2 axis in neointimal hyperplasia after arterial injury in hyperlipidemic mice involves early monocyte recruitment and CCL2 presentation on platelets. Circ Res.

[85] Namiki M, Kawashima S, Yamashita T, Ozaki M, Hirase T, Ishida T, Inoue N, Hirata K, Matsukawa A, Morishita R, Kaneda Y, Yokoyama M (2002). Local overexpression of monocyte chemoattractant protein-1 at vessel wall induces infiltration of macrophages and formation of atherosclerotic lesion: synergism with hypercholesterolemia. Arterioscler Thromb Vasc Biol.

[86] Serbina NV, Pamer EG (2006). Monocyte emigration from bone marrow during bacterial infection requires signals mediated by chemokine receptor CCR2. Nat Immunol.

[87] Fang Y, Shi C, Manduchi E, Civelek M, Davies PF (2010). MicroRNA-10a regulation of proinflammatory phenotype in athero-susceptible endothelium in vivo and in vitro. Proc Natl Acad Sci USA.

[88] Loyer X, Potteaux S, Vion AC, Guerin CL, Boulkroun S, Rautou PE, Ramkhelawon B, Esposito B, Dalloz M, Paul JL, Julia P, Maccario J, Boulanger CM, Mallat Z, Tedgui A (2014). Inhibition of microRNA-92a prevents endothelial dysfunction and atherosclerosis in mice. Circ Res.

[89] Wu W, Xiao H, Laguna-Fernandez A, Villarreal G, Wang KC, Geary GG, Zhang Y, Wang WC, Huang HD, Zhou J, Li YS, Chien S, Garcia-Cardena G, Shyy JY (2011). Flow-dependent regulation of Kruppel-like factor 2 is mediated by microRNA-92a. Circulation.

[90] Zhuang G, Wu X, Jiang Z, Kasman I, Yao J, Guan Y, Oeh J, Modrusan Z, Bais C, Sampath D, Ferrara N (2012). Tumour-secreted miR-9 promotes endothelial cell migration and angiogenesis by activating the JAK-STAT pathway. EMBO J.

[91] Liu D, Zhang XL, Yan CH, Li Y, Tian XX, Zhu N, Rong JJ, Peng CF, Han YL (2014). MicroRNA-495 regulates the proliferation and apoptosis of human umbilical vein endothelial cells by targeting chemokine CCL2. Thromb Res.

[92] Weber KS, Nelson PJ, Grone HJ, Weber C (1999). Expression of CCR2 by endothelial cells : implications for MCP-1 mediated wound injury repair and In vivo inflammatory activation of endothelium. Arterioscler Thromb Vasc Biol.

[93] Kawano S, Nakamachi Y (2011). miR-124a as a key regulator of proliferation and MCP-1 secretion in synoviocytes from patients with rheumatoid arthritis. Ann Rheum Dis.

[94] Tano N, Kim HW, Ashraf M (2011). microRNA-150 regulates mobilization and migration of bone marrow-derived mononuclear cells by targeting Cxcr4. PLoS ONE.

[95] Cheng HS, Sivachandran N, Lau A, Boudreau E, Zhao JL, Baltimore D, Delgado-Olguin P, Cybulsky MI, Fish JE (2013). MicroRNA-146 represses endothelial activation by inhibiting pro-inflammatory pathways. EMBO Mol Med.

[96] Fan W, Fang R, Wu X, Liu J, Feng M, Dai G, Chen G, Wu G (2014). Shear-sensitive microRNA-34a modulates flow-dependent regulation of endothelial inflammation. J Cell Sci.

[97] Liao YC, Wang YS, Guo YC, Lin WL, Chang MH, Juo SH (2014). Let-7g improves multiple endothelial functions through targeting transforming growth factor-beta and SIRT-1 signaling. J Am Coll Cardiol.

[98] Zhan Y, Brown C, Maynard E, Anshelevich A, Ni W, Ho IC, Oettgen P (2005). Ets-1 is a critical regulator of Ang II-mediated vascular inflammation and remodeling. J Clin Invest.

[99] Zhu N, Zhang D, Chen S, Liu X, Lin L, Huang X, Guo Z, Liu J, Wang Y, Yuan W, Qin Y (2011). Endothelial enriched microRNAs regulate angiotensin II-induced endothelial inflammation and migration. Atherosclerosis.

[100] Barish GD, Yu RT, Karunasiri M, Ocampo CB, Dixon J, Benner C, Dent AL, Tangirala RK, Evans RM (2010). Bcl-6 and NF-kappaB cistromes mediate opposing regulation of the innate immune response. Genes Dev.

[101] Barish GD, Yu RT, Karunasiri MS, Becerra D, Kim J, Tseng TW, Tai LJ, Leblanc M, Diehl C, Cerchietti L, Miller YI, Witztum JL, Melnick AM, Dent AL, Tangirala RK, Evans RM (2012). The Bcl6-SMRT/NCoR cistrome represses inflammation to attenuate atherosclerosis. Cell Metab.

[102] Wei Y, Nazari-Jahantigh M, Chan L, Zhu M, Heyll K, Corbalan-Campos J, Hartmann P, Thiemann A, Weber C, Schober A (2013). The microRNA-342-5p fosters inflammatory macrophage activation through an Akt1- and microRNA-155-dependent pathway during atherosclerosis. Circulation.

[103] Yang K, He YS, Wang XQ, Lu L, Chen QJ, Liu J, Sun Z, Shen WF (2011). MiR-146a inhibits oxidized low-density lipoprotein-induced lipid accumulation and inflammatory response via targeting toll-like receptor 4. FEBS Lett.

[104] Michelsen KS, Wong MH, Shah PK, Zhang W, Yano J, Doherty TM, Akira S, Rajavashisth TB, Arditi M (2004). Lack of Toll-like receptor 4 or myeloid differentiation factor 88 reduces atherosclerosis and alters plaque phenotype in mice deficient in apolipoprotein E. Proc Natl Acad Sci USA.

[105] Tian GP, Tang YY, He PP, Lv YC, Ouyang XP, Zhao GJ, Tang SL, Wu JF, Wang JL, Peng J, Zhang M, Li Y, Cayabyab FS, Zheng XL, Zhang DW, Yin WD, Tang CK (2014). The effects of miR-467b on lipoprotein lipase (LPL) expression, pro-inflammatory cytokine, lipid levels and atherosclerotic lesions in apolipoprotein E knockout mice. Biochem Biophys Res Commun.

[106] Maegdefessel L, Spin JM, Raaz U, Eken SM, Toh R, Azuma J, Adam M, Nagakami F, Heymann HM, Chernugobova E, Jin H, Roy J, Hultgren R, Caidahl K, Schrepfer S, Hamsten A, Eriksson P, McConnell MV, Dalman RL, Tsao PS (2014). miR-24 limits aortic vascular inflammation and murine abdominal aneurysm development. Nat Commun.

[107] Kastrup J (2012). Can YKL-40 be a new inflammatory biomarker in cardiovascular disease?. Immunobiology.

[108] Di Gregoli K, Jenkins N, Salter R, White S, Newby AC, Johnson JL (2014). MicroRNA-24 regulates macrophage behavior and retards atherosclerosis. Arterioscler Thromb Vasc Biol.

[109] Vlachos IS, Paraskevopoulou MD, Karagkouni D, Georgakilas G, Vergoulis T, Kanellos I, Anastasopoulos IL, Maniou S, Karathanou K, Kalfakakou D, Fevgas A, Dalamagas T, Hatzigeorgiou AG (2015) DIANA-TarBase v7.0: indexing more than half a million experimentally supported miRNA:mRNA interactions. Nucleic Acids Res 43(database issue):D153–D159. doi:10.1093/nar/gku1215 10.1093/nar/gku1215 PMC4383989 25416803

